# Supplementary figures and images for: Identification and expression analysis of the sucrose synthase gene family in pomegranate (Punica granatum L.)
Source: PeerJ. 2022 Jan 10;10:e12814. doi: 10.7717/peerj.12814 (PMC8757371; doi:10.7717/peerj.12814)

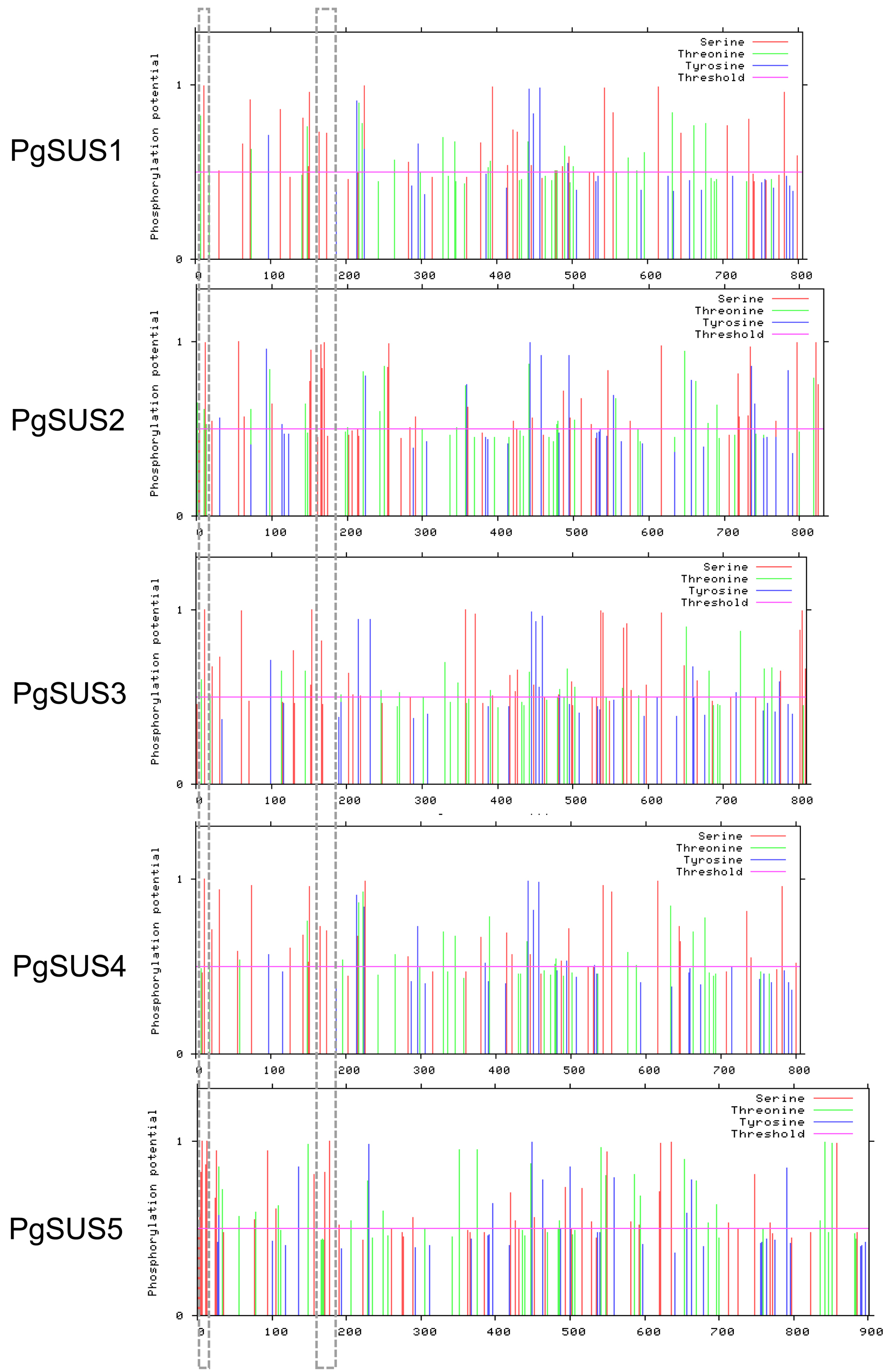

Supplement: Supplemental Information 3 [file peerj-10-12814-s003.jpg]

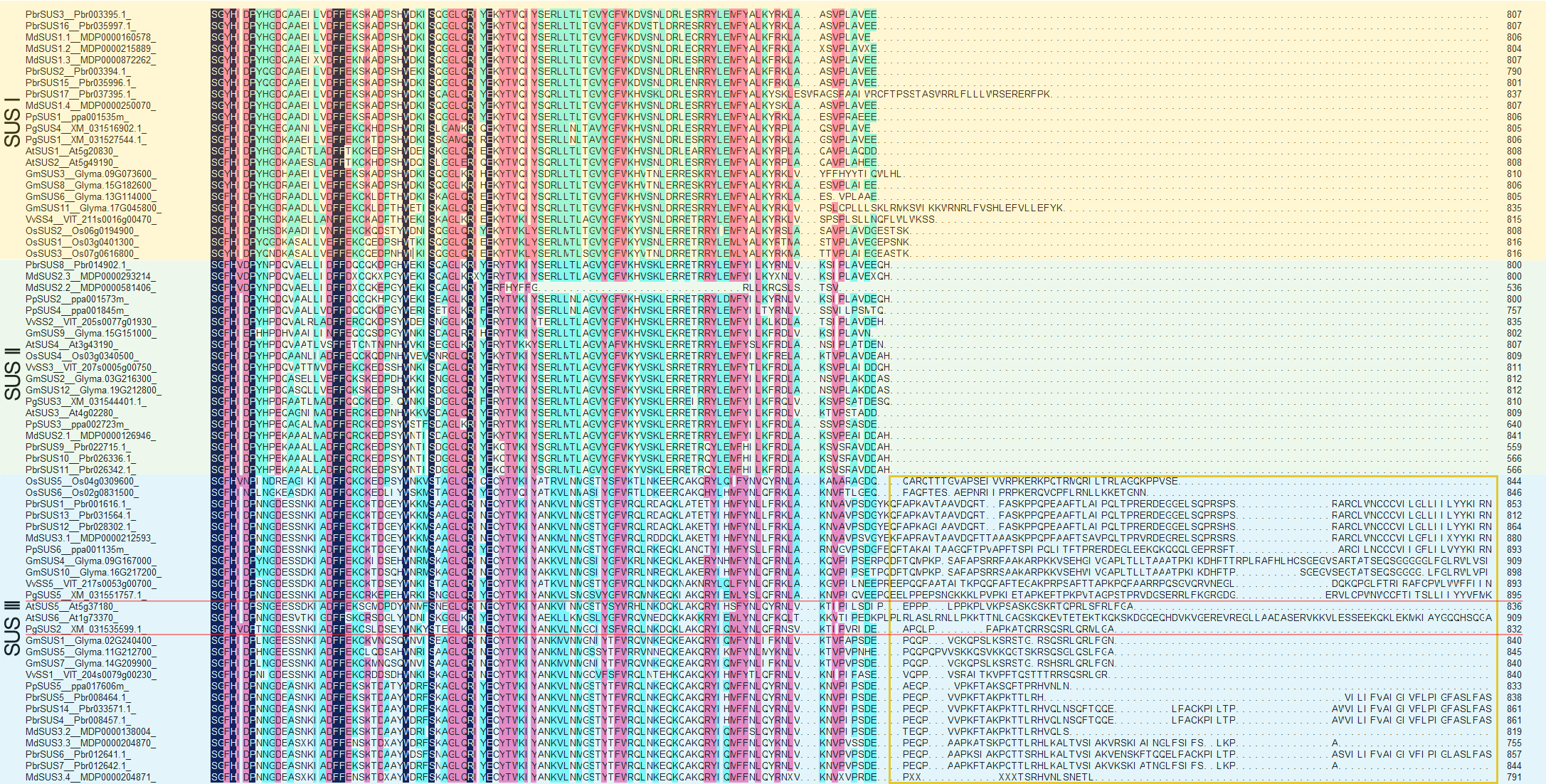

Supplement: Supplemental Information 5 [file peerj-10-12814-s005.jpg]

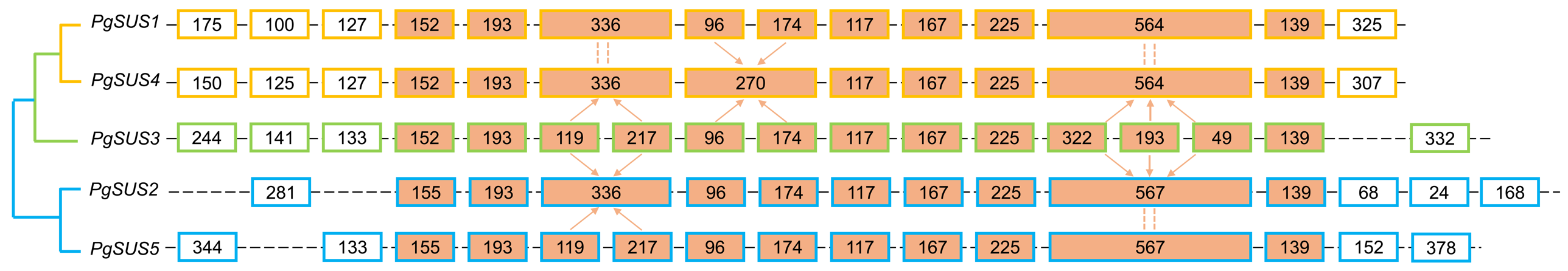

Supplement: Supplemental Information 7 [file peerj-10-12814-s007.jpg]

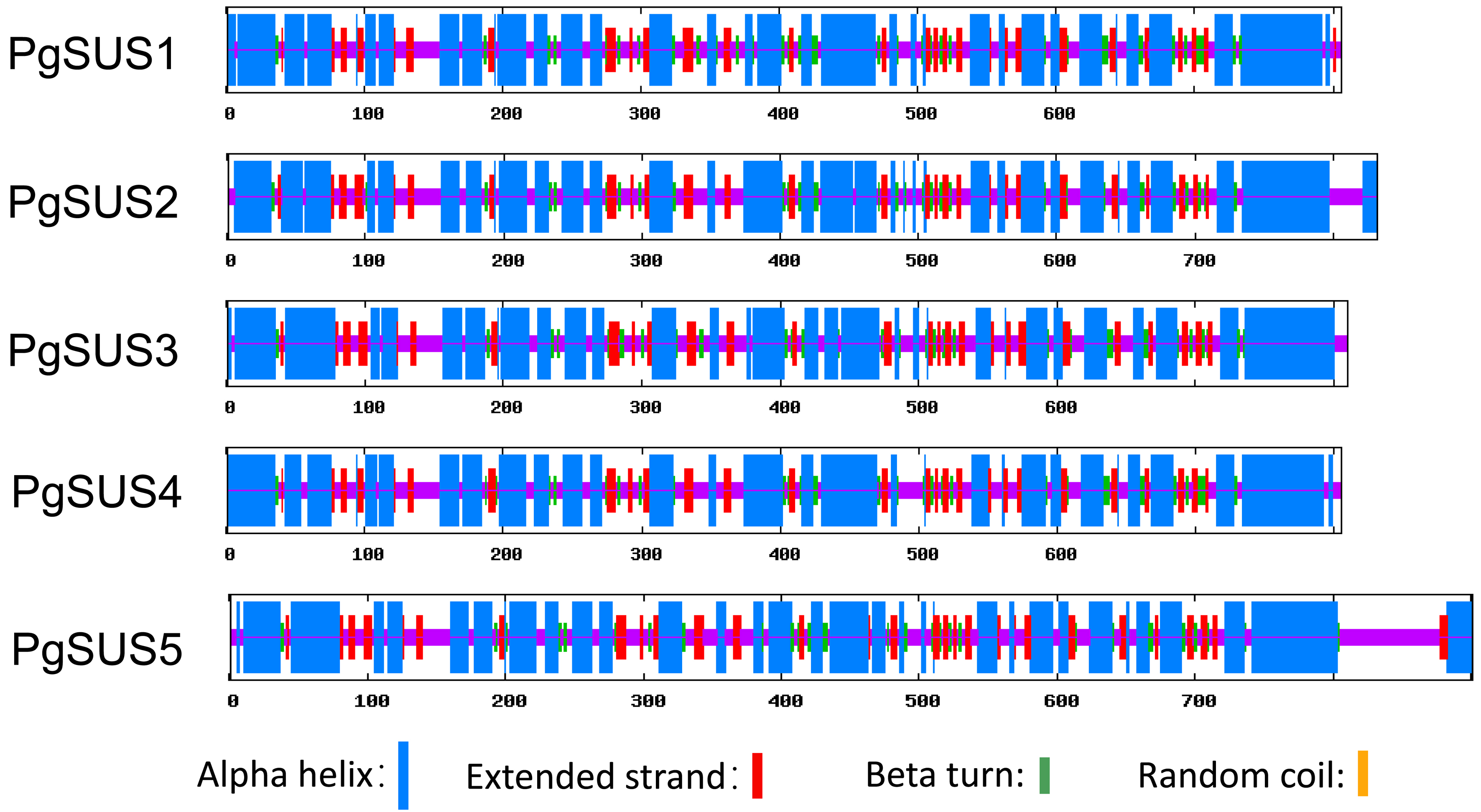

Supplement: Supplemental Information 9 [file peerj-10-12814-s009.jpg]

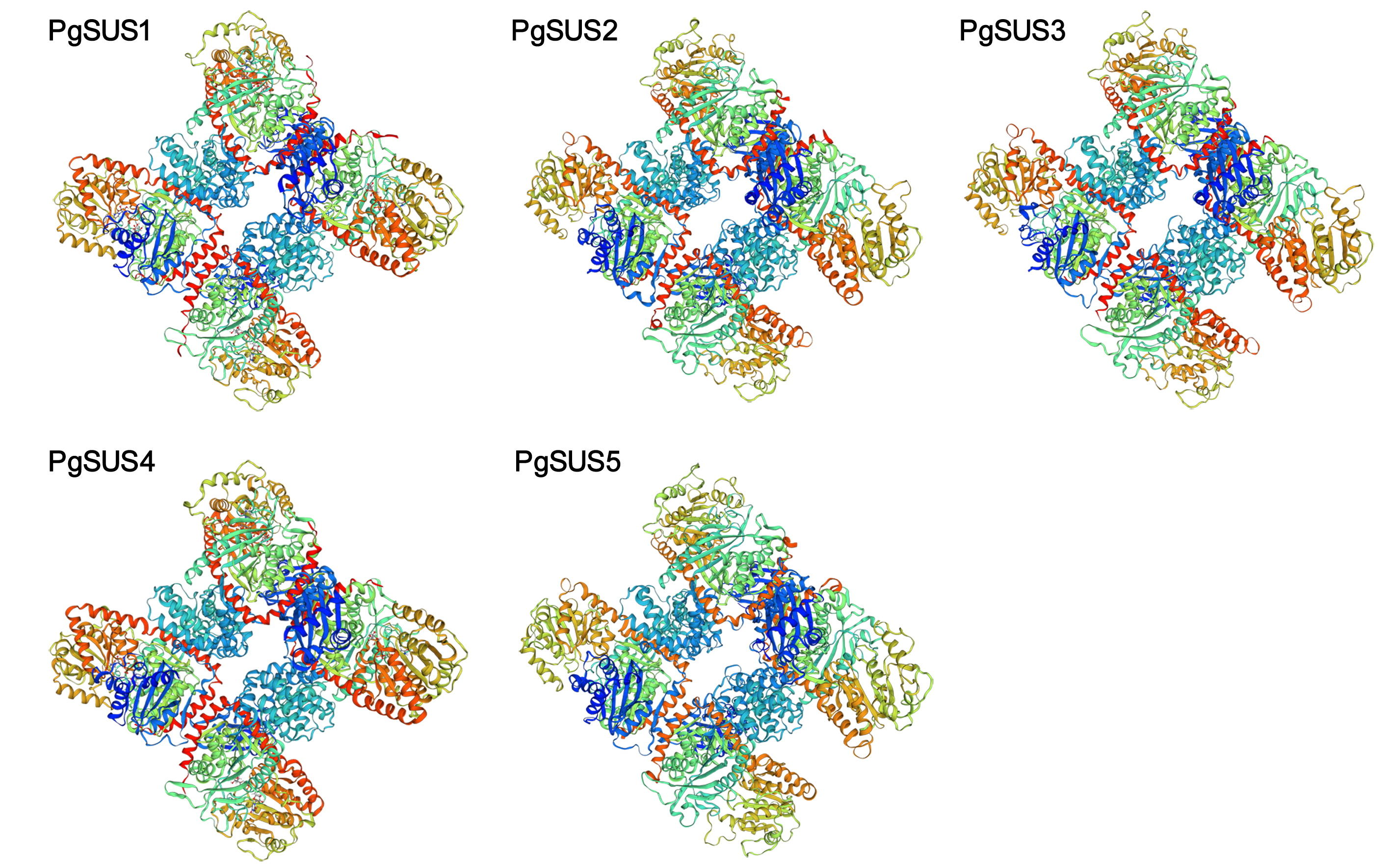

Supplement: Supplemental Information 10 [file peerj-10-12814-s010.jpg]
